# Directed Transfer Function is not influenced by volume conduction—inexpedient pre-processing should be avoided

**DOI:** 10.3389/fncom.2014.00061

**Published:** 2014-06-10

**Authors:** Maciej Kaminski, Katarzyna J. Blinowska

**Affiliations:** Department of Biomedical Physics, Faculty of Physics, University of WarsawWarsaw, Poland

**Keywords:** Directed Transfer Function, volume conduction, multivariate methods of connectivity estimation, pre-processing

The problem of brain connectivity has been gaining more and more interest in the last years. Connectivity can be estimated by different techniques and at the different levels of the hierarchy of the nervous system. Here we shall consider functional connectivity at the level of the brain structures, in particular connectivity measures derived from scalp EEG measurements. A multitude of estimators are being used for connectivity estimation: linear, nonlinear, bivariate and multivariate. The disadvantage of bivariate estimators is connected with the fact, that in virtue of common feeding many spurious connections are found. This fact was demonstrated in works: (Blinowska et al., 2004; Kuś et al., 2004). Let us consider the common situation when a source is emitting activity measured at *N* electrodes. In case of bivariate measures beside *N* true connections also false connections between all electrodes will be find in virtue of common feeding and their number will be [*N*(*N* − 1)/2 − *N*], so they will outnumber the true connections.

This fact directed the interest of the scientific community to the multivariate methods of connectivity estimation. In particular, multivariate estimators based on the Granger causality principle allow to estimate directed connectivity and does not produce spurious connections. Among these estimators widely applied are: Directed Transfer Function (Kamiński and Blinowska, 1991) and Partial Directed Coherence (Baccala and Sameshima, 2001) based on the Multivariate Autoregressive Model (MVAR). Currently DTF and PDC are commonly used and have became a part of various signal processing packages, e.g., eConnectome (http://econnectome.umn.edu), Octave-Forge (http://octave.sourceforge.net/tsa/function/mvfreqz.html), Epilab (http://www.epilepsiae.eu/project_outputs/epilab_software), SIFT (http://sccn.ucsd.edu/wiki/SIFT).

Unfortunately we have noticed that quite often the application to DTF of inappropriate preprocessing routines produces misleading results. Moreover, the use of these routines is not necessary in virtue of the properties of DTF. As the authors of the DTF estimator, we are particularly concerned that the method is applied correctly.

Among the pre-processing methods used before DTF application the most common approach is to project the signals into the source space in order to eliminate volume conduction effect. However, DTF for a pair of channels *i* and *j* is nonzero only, if there is a phase difference between channels *i* and *j*.

The volume conduction is a propagation of the electromagnetic field, so it does not produce a phase difference at electrodes. Therefore, DTF is practically insensitive to volume conduction. The fact that the estimators of connectivity based on the phase difference between channels are not influenced by the volume conduction was also recognized in Stam et al. (2009). The influence of a signal of a constant phase on DTF was demonstrated by means of simulation. For the set of EEG signals we have added a sinusoid, with the same phase for each signal. The amplitude of that sinusoid (of 20 Hz) was similar to the amplitudes of EEG signals. The result is shown in Figure 1. We can observe a prominent peak at 20 Hz in the power spectra of the signals, but this peak is absent in DTF functions.

**Figure 1:**
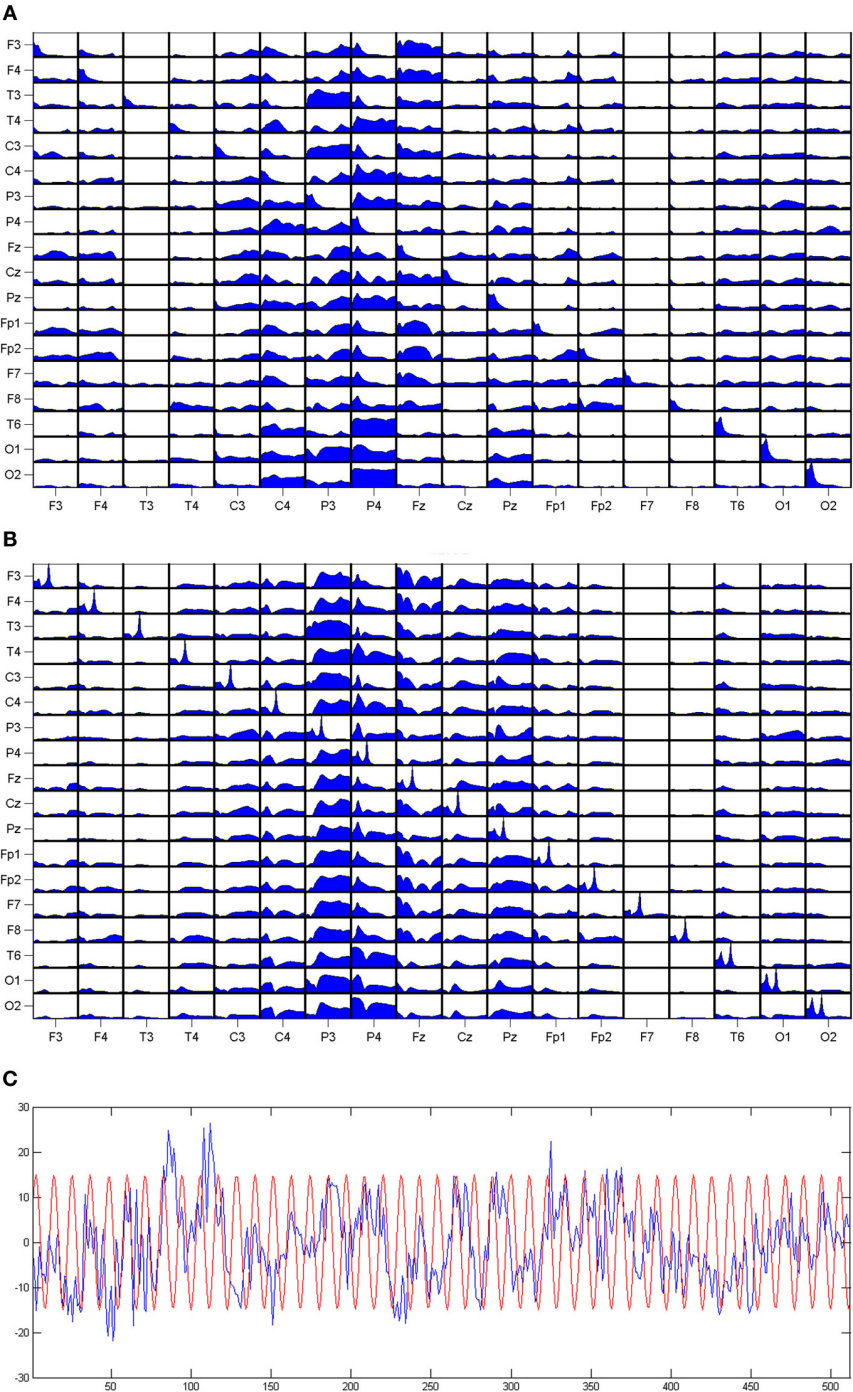
**The DTFs (as functions of frequency) estimated for a set of EEG signals. (A)** The results for original dataset, **(B)** same data with 20 Hz sinusoid added with constant phase to each channel. On the diagonals of the panels power spectra are shown. The propagation from the channel marked below the pictures to the channel marked at the left. At the **(C)** EEG from one channel with superimposed sinusoid.

The fact that DTF is not influenced by volume conduction effects is further supported by the excellent topographical agreement of DTF results with the evidence obtained from anatomical data, imaging studies and physiological experiments. Clear cut patterns of propagation emerged from these studies. As the examples may serve the studies of finger movement (Ginter et al., 2001; Kuś et al., 2006), localization of epileptic focus (Franaszczuk et al., 1994) results obtained for Constant Attention Test (Blinowska et al., 2010) and working memory paradigm (Brzezicka et al., 2010; Blinowska et al., 2013). More examples may be found in Blinowska (2011); Blinowska and Zygierewicz (2011). The animations illustrating dynamically changing patterns of connectivity obtained by the Short-time Directed Transfer Function (SDTF) in some of the above mentioned experiments are available at http://brain.fuw.edu.pl/~kjbli.

Since DTF is immune to volume conduction the pre-processing procedures such as projections on the cortex surface or Laplace transform are not needed. Moreover, they destroy the original correlation structure of the set of signals. If we mix the information from different channels calculating for example Laplacian, we influence the correlation between signals and the information about the phase relations between channels is disturbed. As a result, information on causality or, in another words, on the propagation of activity from channel *j* to *i* is lost.

The results of the works where the preprocessing involving projection on the cortex was applied show a disorganized structure of connectivity. The pre-processing of the data before the DTF application should be limited to subtraction of the mean, possibly division by the variance and digital filtering. However, the filtering must not influence phases of signals. These can be achieved by filtering forward and backward (e.g., Matlab procedure *filtfilt*). The signals should be referenced to the “neutral” derivation, e.g., linked ears, nose, or similar. No common average or bipolar reference may be used. There should be no preprocessing by means Hjorth or Laplace transform, Independent Components Analysis or projection of signals on the cortex. In the view of robustness of DTF in respect to volume conduction, these procedures are obsolete, furthermore, they are harmful and may produce misleading results.

It is worth mentioning that in general methods of connectivity estimation based on phase differences are insensitive to volume conduction. For instance this property holds also for Partial Directed Coherence, but not for ordinary coherence, because it contains common component, which includes activity of no phase difference between electrodes. In conclusion we would like to underline that the preprocessing of the signals for estimation of connectivity should be based on the thorough understanding of the properties of the applied methods.

## Conflict of interest statement

The authors declare that the research was conducted in the absence of any commercial or financial relationships that could be construed as a potential conflict of interest.
